# Assessing keel bone damage in laying hens by palpation: effects of assessor experience on accuracy, inter-rater agreement and intra-rater consistency

**DOI:** 10.3382/ps/pey326

**Published:** 2018-07-26

**Authors:** S Buijs, J L T Heerkens, B Ampe, E Delezie, T B Rodenburg, F A M Tuyttens

**Affiliations:** 1 Animal Sciences Unit, Flanders Research Institute for Agriculture, Fisheries and Food (ILVO), Scheldeweg 68, B-9090 Melle, Belgium; 2 Bristol Veterinary School, Langford House, Langford BS40 5DU, United Kingdom; 3 Agri-Food and Biosciences Institute (AFBI), Large Park, Hillsborough BT26 6DR, United Kingdom; 4 Aeres University of Applied Sciences, De Drieslag 4, 8251 JZ Dronten, The Netherlands; 5 Behavioural Ecology Group, Wageningen University, P.O. Box 338, 6700 AH Wageningen, The Netherlands

**Keywords:** keel bone, fracture, deviation, palpation, accuracy

## Abstract

Accurate assessment is essential when evaluating keel bone damage. Palpation is commonly used to assess keel bone damage in living hens. However, there is little information on the accuracy of assessment of deviations and fractures on different parts of the keel, and on the consistency within, and agreement between, assessors. Crucially, although the importance of experience is commonly emphasized, knowledge on its effect is scarce. Ten assessors with or without prior experience palpated the same 50 75-wk-old hens for deviations, medial fractures, and caudal fractures (scored as present/absent). Accuracy, sensitivity, specificity, precision, and negative predictive value were determined by comparing palpation scores to post-dissection assessment, and then compared between experienced and inexperienced assessors. To determine the effect of the experience gained during the experiment, hens were subsequently re-assessed. Consistency within, and agreement between, assessors were also determined. Assessors with prior experience were more accurate (proportion of accurately assessed deviations: experienced 0.83 vs. inexperienced 0.79±0.01, *P* = 0.04; medial fractures: 0.82 vs. 0.68±0.03 in session 1 only, *P* = 0.04; caudal fractures: 0.41 vs. 0.29±0.03, *P* = 0.03), and inexperienced assessors classified medial fractures more accurately in session 2 (session 1: 0.68 vs. session 2: 0.77±0.04, *P* = 0.04). However, effect sizes were small for deviations and even experienced assessors lacked accuracy when assessing caudal fractures. Unexpectedly, deviations tended to be assessed more accurately in session 1 than in session 2, regardless of assessor status (1: 0.83 vs. 2: 0.79±0.01, *P* = 0.06), suggesting that prolonged assessment contributes to errors. Prior experience decreased specificity and precision of fracture assessment (more unfractured keels were classified as fractured) even though overall accuracy was greater. Intra-rater consistency was fair to good (0.55 to 0.67) for deviations and medial fractures, but poor to fair (0.36 to 0.44) for caudal fractures, and unaffected by prior experience (*P* = 0.49 to 0.89). In conclusion, experience improves accuracy to a limited extent but does not guarantee high accuracy for all types of damage. Future research should determine if other training methods (e.g., comparison to post-dissection scores or to radiographs) improve accuracy.

## INTRODUCTION

Keel bone damage is a major welfare problem facing the laying hen industry due to its high prevalence (up to 97% of hens affected at the end of the laying cycle, Rodenburg et al., 2008). It extends to all housing systems and genetic lines, although to varying degrees (Harlander-Matauschek et al., 2015). There are 2 main types of keel bone damage: fractures and deviations. Fractures are characterized by sharp bends, shearing and/or fragmentation of the keel bone. Deviations are characterized by an abnormally shaped structure that varies from a theoretically perfect 2-dimensional straight plane, or by indentations along the ventral surface, neither being due to fracture (Casey-Trott et al., 2015). Whilst studies on keel bone damage reliably include assessment of fractures, deviations are often disregarded (e.g., Petrik et al., 2013), not discerned from fractures (e.g., Scholz et al., 2008), or only scored if the keel was not fractured (e.g., Stratmann et al., 2015). Although some factors increase the risk of both types of keel bone damage (e.g., loss of structural bone, Pickel et al., 2011), the proximate causes of fractures and deviations are often assumed to differ. Whilst fractures are thought to result from short-term, high energy impact (e.g., collisions with the housing system), deviations likely result from long-term, low energy impact (e.g., pressure on the keel when perching, Pickel et al., 2011; Harlander-Matauschek et al., 2015). The known effects on bird welfare also differ. Even after healing, fractures may cause pain (Nasr et al., 2012a, 2013) and reduce mobility (Richards et al., 2012; Nasr et al., 2012b). The welfare impact of keel bone deviations without fractures is presently unclear (Riber et al., 2018), but it has been suggested that deviations may complicate balancing and lead to unequal bone loading during wing-flapping, increasing the risk of fractures (Harlander-Matauschek et al., 2015).

Because the cause and impact of both types of damage likely vary, it is important to assess these separately when evaluating remedial measures. The most common method to assess both types of damage is by palpation of intact birds. Healing of fractures causes callus formation which can be felt by careful palpation (Wilkins, et al., 2004; Casey-Trott et al., 2015). As calluses take some time to develop studies generally focus on old, healed breaks only. There has been considerable interest in the accuracy of such palpation techniques (i.e., the proportion of samples that are correctly classified as fractured or non-fractured). Acquiring experience with palpation techniques is seen as an essential part of accurate fracture assessment (Casey-Trott et al., 2015). However, the only previous study that compared several assessors with and without prior experience found no difference in their accuracy (Petrik et al., 2013), although assessors without prior experience became more accurate as the experiment progressed (i.e., as they became more experienced). Whilst Petrik et al. (2013) studied the keel bone as a whole, others have discerned between fractures of the medial area and the caudal tip of the keel bone (Casey-Trott et al., 2015; Heerkens et al., 2016). Although the first study found no difference, the second study indicated a lower accuracy for assessment of the caudal tip. Even less information is available on the accuracy of keel bone deviation assessment. This was only evaluated twice, both times within a single observer and estimates varied considerably between these 2 studies (0.91 in Casey-Trott et al., 2015 vs. 0.79 in Heerkens et al., 2016). The role of experience has not yet been investigated for keel bone deviations.

This study aimed to determine how experience prior to, and gained within, an assessment session would influence the accuracy (and other performance statistics) of keel bone damage assessment by palpation. We hypothesized that experienced assessors would be more accurate than inexperienced ones when assessing deviations, medial fractures, and caudal fractures. In addition, we hypothesized that at least the inexperienced assessors would become more accurate as the experiment progressed and they became more familiar with the technique.

## MATERIALS AND METHODS

This experiment involved 10 assessors, 4 of which had previous experience with performing all 3 types of keel bone assessment. Two of these assessors had been trained by an expert assessor as part of a European research project (ERA-net CORE Organic II—HealthyHens). The third assessor had been trained by the first 2, and in turn trained the fourth. Experienced assessors 1 to 3 had each scored approximately 60 flocks in the 2 yr before the experiment, whereas the fourth assessor had scored approximately 20 flocks. On these occasions, 50 to 100 hens were assessed per flock and the assessors regularly compared their scoring to each other to improve agreement. One of these assessors compared palpation scores to post-dissection scores to improve palpation accuracy. This palpation-dissection comparison was only performed on a single occasion. A fifth assessor had been trained by a different international expert to assess medial fractures only, and had subsequently gained experience several years before the experiment by scoring 20 flocks (100 hens/flock) and by making regular comparisons between palpation and post-dissection scores (15 hens/flock). This assessor was considered an experienced assessor for medial fractures only. However, this fifth assessor had performed palpations on a very limited number of hens in the 7 yr prior to the experiment. Therefore, his status as “experienced” was somewhat questionable and the analyses of medial fractures were repeated without this assessor's results. The remaining 5 assessors had no previous experience with keel bone assessment, but all had experience handling poultry. Prior to the experiment all assessors were given a 30-min audio–visual training in which the assessments were explained but no samples were handled.

Intact carcasses of 50 hens (Lohmann Brown Classic) obtained from a commercial aviary system with outdoor access when 75 wk old were frozen until palpation (2 mo later). All birds were thawed and then palpated to assess keel bone deviations, and healed fractures on the medial and caudal tip of the keel bone. Palpations were performed as described by Heerkens et al. (2016): the caudal tip was defined as the last centimetre of the keel bone, the rest of the bone was considered medial, and the dorsal side of the keel bone was not palpated. Each hen was assessed separately by the 10 assessors (to determine the effects of prior experience). When all 50 hens had been assessed by all assessors, the entire assessment process was repeated with an approximately half hour break in between. The second assessment was performed without access to the results of the first assessment (to additionally determine the effects of experience gained within the trial, as well as within-assessor consistency). Keel bone deviations were scored on a binary scale (0: absent, i.e., summed deviation in all directions <0.5 cm, 1: present, i.e., summed deviation in all directions >0.5 cm). Medial and caudal tip fractures were also scored on a binary scale (0: absent, i.e., no callus formation, 1: present, i.e., callus formation). After scoring, the keel bones were roughly excised and frozen, to be cleaned and assessed again later by 1 experienced assessor. This post-dissection assessment was used as the gold standard when determining the accuracy of the palpation technique. The post-dissection assessor had previously participated in the assessment of the intact hens, but was blinded to those results during post-dissection assessment.

### Statistical Analysis

All statistics were performed in R 3.3.3 (R Core Team, 2017). Performance statistics were calculated by comparing the palpation assessment to the true prevalence as indicated by post-dissection assessment. The accuracy (correct assessments/all assessments), sensitivity (true positives/(true positives + false negatives)), specificity (true negatives/(true negatives + false positives)), precision (a.k.a. positive predictive value: true positives/(true positive + false positives)), and negative predictive value (true negative/(true negative + false negatives)) were calculated per assessor per session. Subsequently each of the performance statistics was analysed separately to determine the effects of prior experience, session and their interaction. These analyses were performed using linear mixed models, treating values of the same assessor as repeated measures. Non-significant (*P* > 0.10) interactions and main effects were removed from the models. When interactions occurred, pairwise comparisons were made using step-up Bonferroni correction. Pairs differing in 2 factors were not compared (e.g., experienced assessors in session 1 vs. inexperienced assessors in session 2).

Inter-rater agreement was evaluated using Fleiss–Cuzick Kappa values calculated separately for experienced and inexperienced assessors in each session. Intra-rater consistency was assessed by calculating Cohen's Kappa for each assessor and subsequently assessing the effect of prior experience on these Kappa values in a linear model. Values were interpreted according to Cicchetti (1994): <0.4 poor, 0.4 to 0.6 fair, 0.6 to 0.75 good, and >0.75 excellent. Although the use of Kappa statistics to assess agreement has been criticized by some, the alternatives are not undisputed either (Cicchetti et al. 2017) and/or require normally distributed data, and therefore Kappa based statistics were deemed most suitable in this case.

## RESULTS

### Deviations

**Figure 1.**
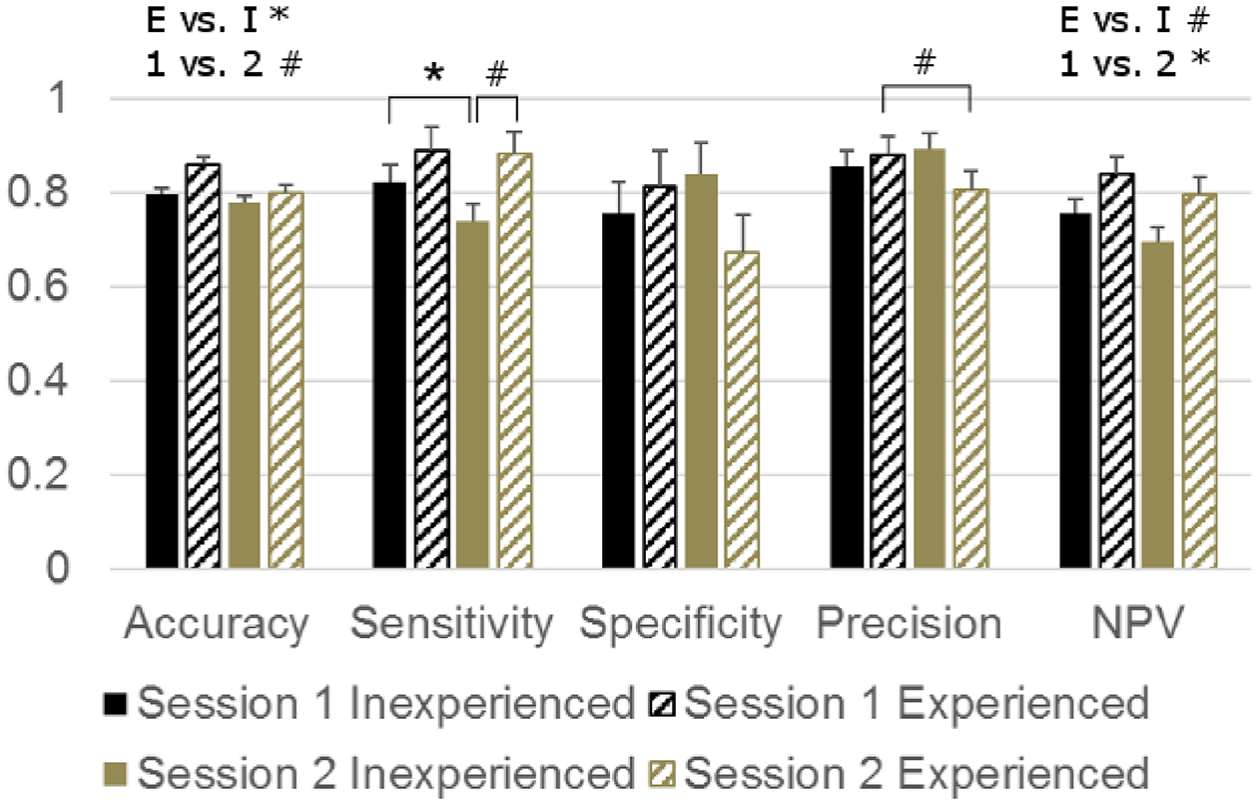
LSMEANS + SEM of performance statistics for binary keel bone deviation assessment (absent: deviation <0.5 cm, present: deviation >0.5). E vs. I: main effect of experience (E) vs. inexperience (I) prior to the experiment, 1 vs. 2: main effect of session, 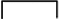: interactive effect, pairs connected by the line (tend to) differ after step-up Bonferroni adjustment (pairs differing in 2 factors not compared). *P < 0.05, # *P* < 0.10. NPV = negative predictive value.

The true prevalence of keel bone deviations was 60%, close to the percentage classified as deviated by our experienced assessors (average 64%, min–max: 56 to 68). On average, our inexperienced assessors were also close to the true prevalence, although their range was wide (average 55%, min–max: 39 to 70). Performance of the binary classification was good (>0.68) according to all statistics in both sessions and for both inexperienced and experienced assessors (Figure 1). However, some differences between the sessions and groups occurred. Inexperienced assessors were slightly less accurate than experienced assessors when assessing deviations (i.e., were more likely to misclassify hens, *P* = 0.042). Also, assessment tended to be less accurate in the second session than in the first (*P* = 0.060). Sensitivity, specificity, and precision were affected by an experience*session interaction (*P* = 0.040, *P* = 0.050, and *P* = 0.032, respectively). Inexperienced assessors were less sensitive in the second than in the first session (i.e., classified more deviated keels as non-deviated). Within this session they also tended to be less sensitive than experienced assessors. The interactive effect on specificity (i.e., the proportion of non-deviated keels that were classified correctly) did not result in significant pairwise differences. The precision of the experienced assessors tended to be lower in the second than in the first session (i.e., fewer keels that were classified as deviated were truly deviated). Negative predictive value tended to be lower in inexperienced assessors and was significantly lower in the second session (i.e., fewer keels that were classified as non-deviated were truly non-deviated, *P* = 0.064 and *P* = 0.006, respectively).

Inter-rater agreement of deviation scoring during the first session was good for experienced assessors but poor for inexperienced assessors (i.e., different experienced assessors more often gave a keel the same classification than different inexperienced assessors did, Table 1). It was fair for both types of assessor in the second session. Intra-rater consistency (the proportion of samples receiving the same score when reassessed by the same assessor corrected for chance) was fair to good and did not differ significantly between experienced and inexperienced assessors (Table 2).

**Table 1.** Inter-rater agreement as indicated by Fleiss–Cuzick Kappa values for experienced and inexperienced assessors in both sessions. CI = confidence interval.

Assessment	Session	Assessor	Fleiss–Cuzick Kappa (CI)	Interpretation
Deviations	1	Inexperienced	0.39 (0.23 to 0.54)	Poor
		Experienced	0.65 (0.50 to 0.80)	Good
	2	Inexperienced	0.49 (0.35 to 0.64)	Fair
		Experienced	0.45 (0.27 to 0.63)	Fair
Medial fractures	1	Inexperienced	0.50 (0.35 to 0.66)	Fair
		Experienced	0.38 (0.17 to 0.59)	Poor
		Experienced excl. assessor 5	0.50 (0.26 to 0.75)	Fair
	2	Inexperienced	0.48 (0.31 to 0.65)	Fair
		Experienced	0.35 (0.13 to 0.56)	Poor
		Experienced excl. assessor 5	0.53 (0.27 to 0.79)	Fair
Caudal tip fractures	1	Inexperienced	0.22 (0.00 to 0.43)	Poor
		Experienced	0.28 (0.11 to 0.45)	Poor
	2	Inexperienced	0.24 (0.00 to 0.49)	Poor
		Experienced	0.37 (0.20 to 0.54)	Poor

**Table 2.** Intra-rater consistency (proportion of samples receiving the same score when reassessed by the same assessor) for experienced and inexperienced assessors.

Assessment	Assessor	LSMEAN	SEM	*P*-value
Deviation	Inexperienced	0.55	0.05	0.485
	Experienced	0.61	0.06	
Medial fractures	Inexperienced	0.65	0.07	0.881^1^
	Experienced^1^	0.67^1^	0.07^1^	
Caudal tip fractures	Inexperienced	0.36	0.08	0.552
	Experienced	0.44	0.10	

^1^LSMEANS ± SEM for experienced assessors after removal of assessor 5 (because of ambiguous experience status) were 0.65 ± 0.09 and were not found to differ from inexperienced assessors (*P* = 0.956).

### Medial Fractures

The true prevalence of healed medial fractures was 88%. Flock level prevalence as assessed by experienced assessors was close to the true prevalence and showed a narrow range (average 86%, min–max 81 to 89) after the exclusion of assessor 5. All inexperienced assessors on the other hand underestimated this prevalence and their range was relatively wide (average 63%, min–max 55 to 76%).

**Figure 2.**
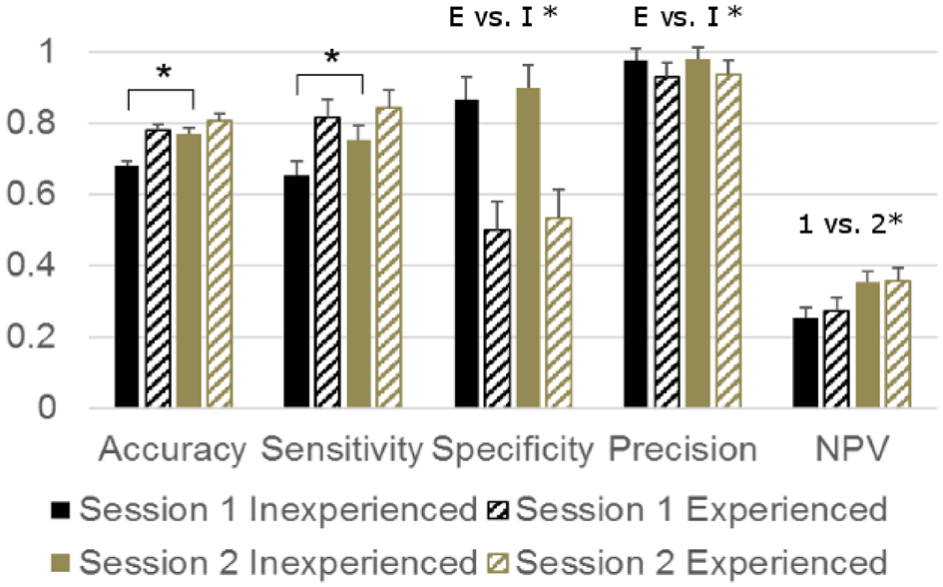
LSMEANS + SEM of performance statistics for binary assessment of healed medial keel bone fractures (absent: no callus formation, present: callus formation). E vs. I: main effect of experience (E) vs. inexperience (I) prior to the experiment, 1 vs. 2: main effect of session, 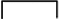: interactive effect, pairs connected by the line differ after step-up Bonferroni adjustment (pairs differing in 2 factors not compared). *P < 0.05. NPV = negative predictive value.

As our sample mainly included fractured keels, accuracy, sensitivity, and precision mainly (or wholly) depended on the correct classification of truly fractured keels. The good to very good (0.65 to 0.93) scores for these 3 statistics show that our assessors had relatively little difficulty classifying the fractured keels. They had more problems exclusively classifying the small percentage of truly unfractured keels as such, as reflected by the lower scores for specificity (<0.54) for experienced assessors and the poor negative predictive value (<0.36) for all groups (Figure 2).

Accuracy and sensitivity were affected by an experience*session interaction (*P* = 0.036 and 0.019, respectively). The improvement in both statistics between the first and second session reached significance for inexperienced assessors only (i.e., inexperienced assessors classified more keels correctly overall, and also classified more fractured keels correctly, during the second as compared to the first session). Specificity and precision were higher in inexperienced assessors than in experienced ones (i.e., experienced assessors classified more unfractured keels as fractured relative to the number of keels they correctly classified as either fractured or unfractured, *P* = 0.030 and *P* = 0.047, respectively). Negative predictive value was higher in the second session (i.e., a smaller proportion of the keels that were classified as unfractured were actually fractured, *P* = 0.022).

Analysis of the medial data without the fifth experienced assessor (who had an accuracy of 0.64 in both sessions) led to similar outcomes, although more pairwise differences occurred (Table 3). Notably, this meant that during the first session inexperienced assessors had a significantly lower accuracy than experienced assessors, and in both sessions their sensitivity was lower than that of experienced assessors.

**Table 3.** LSMEANS + SEM of performance statistics for binary assessment of healed medial keel bone fractures after removal of assessor 5 because of an ambiguous experience status. NPV = negative predictive value.

	Session 1		Session 2	
	Inexperienced	Experienced	Inexperienced	Experienced
Accuracy	0.68 ± 0.03^a^	0.82 ± 0.03^b^	0.77 ± 0.03^b^	0.85 ± 0.03^b^
Sensitivity	0.65 ± 0.03^a^	0.88 ± 0.03^b,c^	0.75 ± 0.03^b^	0.91 ± 0.03^c^
	Inexperienced		Experienced	
Specificity	0.88 ± 0.05^b^		0.40 ± 0.06^a^	
Precision	0.98 ± 0.01^b^		0.92 ± 0.01^a^	
	Session 1		Session 2	
NPV	0.27 ± 0.02^a^		0.37 ± 0.02^b^	

LSMEANS in the same row lacking a common superscript differ significantly (*P* < 0.05).

Inter-rater agreement of medial fracture scoring was fair between inexperienced assessors, but poor between experienced ones in both sessions (Table 1). However, inter-rater agreement of experienced assessors excluding assessor 5 was fair. Intra-rater consistency was good for both experienced and inexperienced assessors, without a significant difference between these 2 types of assessors (regardless of whether assessor 5 was included, Table 2).

### Caudal Tip Fractures

**Figure 3.**
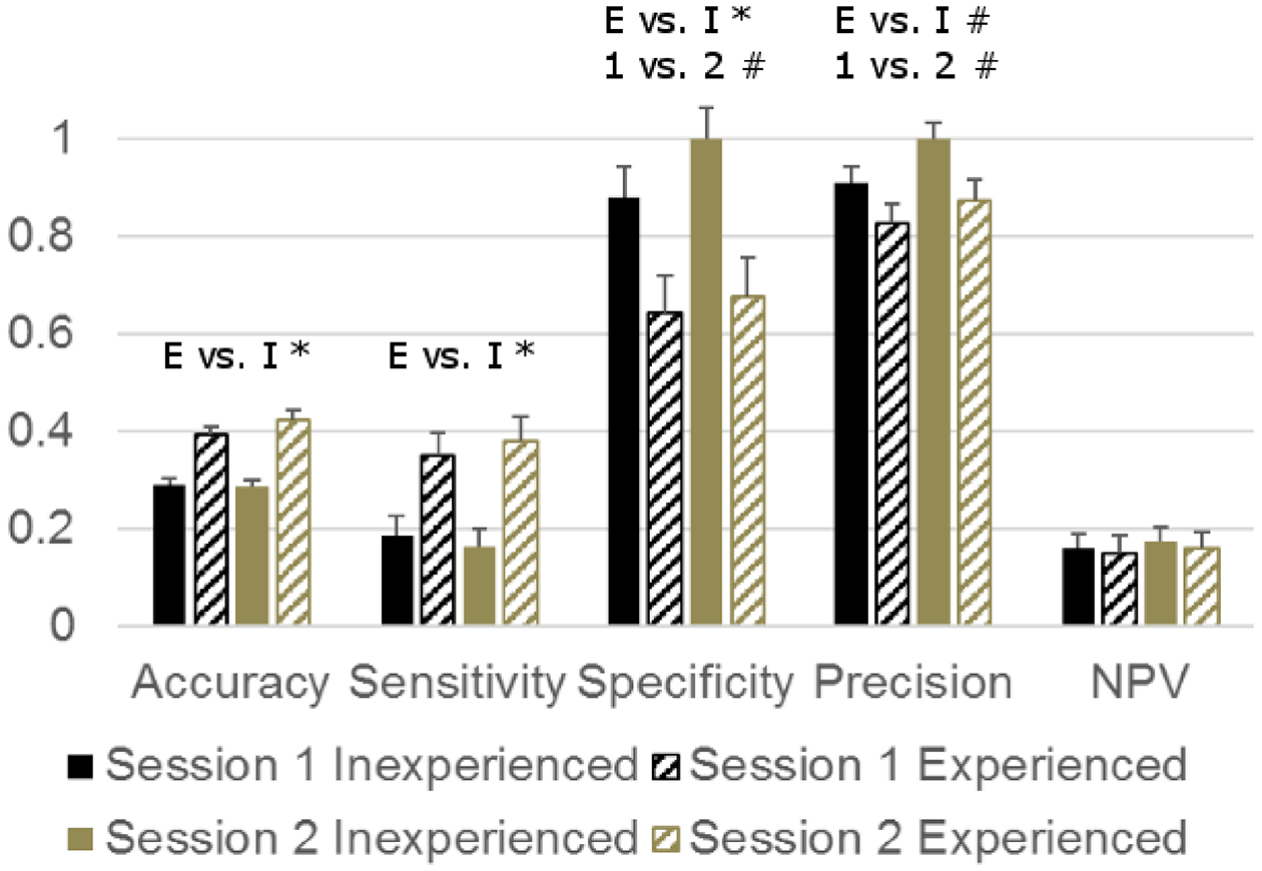
LSMEANS of performance statistics for binary assessment of healed caudal keel bone fractures (absent: no callus formation, present: callus formation). E vs. I: main effect of experience (E) vs. inexperience (I) prior to the experiment, 1 vs. 2: main effect of session, *P < 0.05, # *P* < 0.10. NPV = negative predictive value.

The true prevalence of caudal tip fractures was 85.1%. Hens were often found to have caudal as well as medial fractures (68% of all hens). All assessed hens had either a caudal fracture or a medial one. Our experienced and inexperienced assessors respectively classified only 37% (min–max: 34 to 40%) and 16% (min–max: 4 to 25%) as having a caudal fracture, thus underestimating prevalence markedly. In line with this accuracy, sensitivity and the negative predicted value were poor (0.15 to 0.43, Figure 3), i.e., of the high number of fractured keels in the sample many went undetected. In contrast, specificity and precision were good to perfect (0.64 to 1), i.e., unfractured keels were rarely misclassified as fractured. Experienced assessors had a higher accuracy and sensitivity than inexperienced ones (i.e., made fewer misclassifications in general and classified fewer fractured keels as unfractured, *P* = 0.031 and *P* = 0.005, respectively). However, experienced assessors also had a lower specificity and tended to have a lower precision (i.e., classified more unfractured keels as fractured relative to the number of correctly classified fractured and unfractured keels, *P* = 0.008 and *P* = 0.057, respectively). This misclassification of fractured keels tended to be less common in the second session, as shown by increased specificity and precision in session 2 (both *P* = 0.051). The negative predicted value (the proportion of keels classified as unfractured that were classified correctly) was not affected by experience or session (*P* > 0.10).

Inter-rater agreement of caudal tip fracture scoring was poor, both between experienced and between inexperienced assessors (Table 1). Intra-rater consistency was poor to fair for both types of assessors, without a significant difference between these 2 types (Table 2).

## DISCUSSION

We determined how the quality of keel bone assessment by palpation was affected by experience gained prior to and during the experiment. This was done in a sample with a high true prevalence of deviations (60%) as well as fractures (medial: 88%, caudal: 85%). As all our hens had at least 1 type of keel bone fracture we did not evaluate the accuracy of the pooled keel bone fractures (medial and caudal), as the impossibility of true negatives and false positives would distort the results.

Palpation is an indispensable technique to assess keel bone damage in situations where other techniques like dissection or radiography are impossible or highly impractical. Although palpation accuracy was not perfect, we found that not only extensive but even minimal experience (i.e., assessing 50 hens) with palpation techniques can lead to a relatively accurate assessment of deviations and medial fractures. The technique also showed good consistency within assessors, flock level prevalence estimates close to true prevalences, and fair inter-assessor agreement (at least after the first session). This supports the continued use of palpation for deviations and medial fractures in cases where other methods are not feasible. The wide range in individual inexperienced assessors’ flock level deviation prevalence estimates (and their poor inter-assessor agreement in the first session) suggest that lack of experience can lead to both underestimation and overestimation, depending on the individual assessor. In contrast to the favourable results for deviations and medial fractures, both experienced and inexperienced assessors were inaccurate when assessing caudal tip fractures. They markedly underestimated their prevalence, showed poor agreement between assessors and a consistency within assessors that was only just fair. This suggests that, unless different training methods can improve the accuracy of caudal tip palpation, this technique cannot be recommended for use.

### Deviations

Both types of assessors scored deviations with high accuracy (0.78 to 0.86) in both sessions. But as expected, experience prior to the experiment improved this accuracy. More specifically, experienced assessors had a higher negative predictive value overall and a higher sensitivity in the second session, showing they were less likely than inexperienced assessors to overlook deviations. Although inexperienced assessors’ accuracy of deviation assessment was high and their intra-rater consistency fair, their inter-rater agreement was poor in the first session, showing that different inexperienced assessors made different mistakes. Experienced assessors on the other hand combined a high accuracy and consistency with a good inter-rater agreement in the first session. Somewhat surprisingly, their inter-rater agreement was lower in the second session. This may mean that they were reverting to their own style of palpating or their original training (which was likely somewhat different for each assessor as they had been trained by different trainers at different times). Alternatively, it may mean that the experienced assessors lost their concentration after prolonged scoring. Such an effect seems to have affected the inexperienced assessors as well. Their performance was expected to increase in the second session (as they were now somewhat more experienced). However, the opposite was found: during the second session accuracy tended to be lower. More specifically, inexperienced assessors were less sensitive during the second session (i.e., more deviations were overlooked), whereas experienced assessors were less precise (i.e., more keels that were classified as deviating were actually straight). In addition, both types of assessor had a lower negative predictive value in the second session (i.e., a greater proportion of the keels classified as straight were actually deviated). For inexperienced assessors, it seems probable that their recollection of the training prior to the first session was diminished during the second session. However, this seems less likely for experienced assessors (as their assessment should not be as strongly influenced by the training which was only a reminder for them). Decreased concentration after scoring many hens may also have contributed to the lower accuracy of both types of assessor in the second session. Taken together, these findings suggest that although deviation assessment by palpation is a technique that can be applied reliably after a short training, it can be improved by increased experience. Furthermore, the lower accuracy in the second session suggests that both experienced and inexperienced assessors may benefit from (reminder) training just before starting an assessment session or from avoiding prolonged assessments.

The accuracy of our experienced assessors' keel bone deviation assessment (0.80 to 0.86) was in between the values reported in previous studies using a single experienced assessor (Heerkens et al., 2016: 0.70; Casey-Trott et al., 2015: 0.91). We observed a similar sensitivity and NPV as Casey-Trott (0.88 to 0.89 vs. 0.84, and 0.80 to 0.84 vs. 0.85, respectively), but our specificity and precision were lower (0.68 to 0.81 vs. 0.97 and 0.81 to 0.88 vs. 0.98, respectively), showing that in our study straight keel bones were more often categorized as deviated. Apart from personal skill and the sample that was assessed, this may be because Casey-Trott et al. (2015) used a slightly different way of deviation assessment (in contrast to Casey-Trott et al., in our study deviations in different directions were summed).

### Medial Fractures

In line with our hypothesis, but in contrast to Petrik et al. (2013) we found that prior experience increased the accuracy of medial fracture assessment. This effect was limited to the first session and only became significant after exclusion of the fifth assessor, who had not performed palpations regularly in the years before the experiment (visual analysis before exclusion suggested a similar trend, but this did not reach significance). This contrast with prior research may result from differences between the level of experience of “experienced assessors” in the 2 studies: while all four of our (non-excluded) experienced assessors had assessed keel bone fractures and deviations regularly in the 2 yr prior to the experiment, Petrik's experienced assessors are mentioned to “routinely evaluate keel bone integrity, although not fractures per se.” Surprisingly, some performance statistics were better for our inexperienced than for our experienced assessors: they classified fewer keels without a medial fracture as fractured, which led to a much higher specificity and a somewhat higher precision. The effect was more pronounced for specificity because of the high true prevalence of medial fractures. Specificity is defined as true negatives/(true negatives + false positives), and in a sample with a high true prevalence the number of true negatives as well as false positives is severely limited. However, when a false positive does occur this will have a pronounced effect on specificity. In contrast, precision is defined as true positives/(true positive + false positives), and in a sample with a high true prevalence there is a lot of scope for true positives to occur, but not false positives. Thus, false positives will have only a limited effect on precision. Especially for precision this means that more exact estimates could be possibly be gained from using a sample with a lower true prevalence. However, the high prevalence of the current sample is in line with the prevalence that has been reported for commercial systems (Rodenburg et al., 2008), and the specificity and precision we obtained from this sample are therefore relevant.

It needs to be noted that, within the group of experienced assessors, specificity and precision varied greatly. This suggests that it is not inevitable that experience will increase the chance of false positives, but that this should be a topic of interest during refresher trainings. Both inexperienced and experienced assessors showed a good intra-rater consistency when assessing medial fractures, and (after exclusion of the 5th assessor) inter-rater agreement between the experienced assessors was fair. Before his exclusion inter-rater agreement had been poor (showing that this assessor scored markedly different than the others) and combined with his relatively low accuracy, this could suggest that his palpation skills had decreased due to lack of recent practice. Alternatively, it may indicate that for some assessors even rigorous training and extensive experience may not be sufficient to assure reliable application of keel bone palpation. In either case, this shows that regular feedback on palpation accuracy is of importance.

In line with our other hypothesis that inexperienced assessors would improve during the experiment, their accuracy of medial fracture assessment was higher in the second session. Petrik et al. (2013) found a similar effect of within-experiment experience for inexperienced assessors when assessing medial and caudal fractures as one, although in the absence of an effect of prior experience. Our inexperienced assessors classified fewer fractured keels as non-fractured in the second session, resulting in an improved sensitivity and negative predictive value. Together, these findings suggest that experience with medial fracture assessment improves its accuracy, but only to a limited extent and it may also increase the risk of specific errors (i.e., classifying keels without a medial fracture as fractured). Most of our experienced assessors had acquired experience by applying the technique and comparing amongst each other, rather than by regular comparisons to more accurate methods of assessment (e.g., post-dissection scores or radiological examination) and the 1 assessor who had regularly engaged in such comparisons had done so several years ago. For experience to truly improve the accuracy of medial fracture assessment, it may be necessary to engage in regular comparisons between their palpation score and more accurate methods. Comparison to post-dissection scores will often be the easiest way to do this. However, comparing to radiological assessment would have the added benefit that the fracture could first be located on the radiograph, allowing the assessor to search for it by palpating the right location. This may aid in practicing harder to detect fractures. Although such training methods are certainly also preferable for inexperienced assessors, practicing palpation techniques alone can improve their skill somewhat.

The accuracy of our experienced assessors medial fracture assessment (0.82 to 0.85) was slightly higher than the 0.74 previously reported by Heerkens et al. (2016), but substantially lower than the near-perfect accuracy (0.99) reported in the single assessor study by Casey-Trott et al. (2015). The sensitivity and precision of our experienced assessors were similar to those reported by Casey-Trott et al. (2015), but their specificity and negative predictive value were clearly lower, suggesting a problem with correctly identifying non-fractured keels in our experienced assessors.

### Caudal Tip Fractures

With an accuracy of 0.29 to 0.43 caudal tip fractures were clearly more difficult to assess correctly than medial fractures, in line with previous results by Casey-Trott et al. (2015), although in contrast to Heerkens et al. (2016) who found equal accuracies for both parts. As we had expected, experienced assessors were more accurate and sensitive (i.e., overlooked fewer of the caudal fractures). However, as previously remarked for medial fractures, experienced assessors were also more likely to classify a keel without a caudal tip fracture as if it was fractured, leading to decreased specificity and precision. Intra-rater consistency was poor for inexperienced assessors, and together with the low accuracy this indicates that they made different errors in the 2 sessions. Although numerically slightly higher in experienced assessors, and above the threshold for fair consistency, no evidence was found that experienced assessors were significantly more consistent when scoring caudal tip fractures. Inter-rater agreement was poor for both assessor types and sessions, showing that different assessors made different errors. This is somewhat surprising for the experienced assessors, as they had regularly compared their scoring to each other prior to the experiment, and would thus be expected to show a considerable agreement. This again emphasizes the difficulty of detecting fractures of the caudal tip by palpation. Although both types of assessors were less likely to classify keels without a caudal tip fracture as if they were fractured during the second session, this did not result in a significant improvement of overall accuracy (possibly because the chance of making such an error was small in our sample in which caudal tip fractures had a true prevalence of 85%).

The low sensitivity and negative predictive value show that many caudal fractures were not detected, which would lead to an underestimation of the prevalence if the palpation method is applied without further post-dissection assessment (as is usually the case in routine assessments). In line with this, the flock level prevalence was markedly underestimated by experienced as well as inexperienced assessors (true prevalence: 85%, experienced assessors estimate 37%, inexperienced assessors estimate 16%). However, the accuracy of caudal tip fracture assessment by our experienced assessors (0.39 to 0.43) was much lower than in previous studies (Casey-Trott et al., 2015: 0.88; Heerkens et al., 2016: 0.74). The difference between the studies may stem from the skill of the assessors as well as the ease of detection of fractures (which may depend on how neatly the breaks had healed and the conformation of the hens). As our experienced assessors had mostly gained experience by applying the palpation technique and comparing amongst themselves, this suggests that such training methods may not suffice to assess caudal tip fractures accurately, and may lead to an underestimation of their prevalence. The effects of other, possibly more effective training methods (e.g., regular comparisons to post-dissection scores or radiological examination) require future evaluation.

## Conclusion

Prior experience acquired by applying keel bone palpation techniques improved the accuracy of deviation and fracture assessment, but often only to a small extent. Although accuracy was higher, prior experience increased the likelihood of false positives when assessing fractures (i.e., classifying an unfractured keel as fractured). More rigorous training methods (e.g., comparisons between palpation and highly accurate methods like post-dissection or radiological assessment) may be preferable to the now commonly used method of consensus training (i.e., comparing and discussing palpation scores between assessors). The benefit of such methods should be evaluated in future studies. When assessing medial fractures, assessors lacking any prior experience may benefit somewhat from applying palpation techniques to a test set before doing real assessments, as this can improve their accuracy. However, an opposite effect was found for deviations (possibly due to loss of concentration). Assessment of caudal tip fractures was inaccurate even when performed by experienced assessors and, unless this can be improved by other training methods, it cannot be recommended for use.
